# Changes in Facial Soft Tissue Asymmetry in Class II Patients One Year After Orthognathic Surgery

**DOI:** 10.3390/jcm14092912

**Published:** 2025-04-23

**Authors:** Edmonda Nike, Oskars Radzins, Ville Vuollo, Anda Slaidina, Andris Abeltins

**Affiliations:** 1 Department of the Orthodontics, Institute of Stomatology, Rīga Stradiņš University, LV-1007 Riga, Latvia; andris.abeltins@rsu.lv; 2 Baltic Biomaterials Centre of Excellence, Institute of Stomatology, Rīga Stradiņš University, LV-1007 Riga, Latvia; 3 Research Unit of Population Health, Faculty of Medicine, University of Oulu, 90570 Oulu, Finland; 4 Medical Research Center, Oulu University Hospital, University of Oulu, 90220 Oulu, Finland; 5 Department of Prosthodontics, Institute of Stomatology, Rīga Stradiņš University, LV-1007 Riga, Latvia; anda.slaidina@rsu.lv

**Keywords:** angle Class II, facial asymmetry, malocclusion, orthodontics, orthognathic surgery, stereophotogrammetry

## Abstract

**Objectives:** The objective of this study was to examine changes in facial soft tissue asymmetry over time in patients after Class II orthognathic surgery using three-dimensional (3D) stereophotogrammetry. **Methods**: The study consists of 54 patients with a skeletal Class II malocclusion (32 female, 22 male; mean age, 33.2 years). Three-dimensional photographic data were collected using the 3dMD Trio stereophotogrammetry system. The evaluation of 21 anthropometric landmark positions was conducted before surgery (T0), 6 months (T1), and 12 months (T2) after surgery. Facial asymmetry was classified as mild (0–2 mm), moderate (3–5 mm), or severe (>5 mm). **Results**: There was a small difference in the mean distance when analyzing the asymmetry of the whole face. The 3D measurements showed statistically significant differences (*p* < 0.05) between T0 versus T1 and T2 time-point values. Prior to surgery, males exhibited a higher degree of soft tissue asymmetry compared to females. The chin volume asymmetry score was higher in the females of the cohort and patients undergoing bimaxillary surgery (median 1.11) than in the males of the cohort and patients undergoing single-jaw surgery (median 1.08); however, these differences were not statistically different. **Conclusions**: The findings indicate that soft tissue asymmetry may become altered within a 6-month period following surgery.

## 1. Introduction

Asymmetry of the face is a three-dimensional (3D) problem. It is often seen in patients who have dentofacial deformities or malocclusions [1,2]. Asymmetry can significantly affect facial appearance. As a result, the psychosocial quality of life is compromised, particularly for patients with moderate-to-severe asymmetries. Much more attention is being paid to soft tissue, which can have a more direct impact on esthetics than hard tissue [3].

Worldwide prevalence of facial asymmetry ranges from 11% to 44% [3,4] and has been noted to range from 21% to 85% [5]. It is important to remember that absolute facial symmetry does not exist. In fact, there is a range of facial asymmetry that is considered to be acceptable [3,4,6,7].

Orthognathic surgery is an approach used for many years to treat adults with Class II severe skeletal malocclusion. The goal of this intervention is to obtain a more harmonious relationship between the facial bones during functional occlusion and improve esthetics [4,8,9]. A well-proportioned chin can make a significant contribution to the harmony of the face, especially when viewed in profile [2,6,8].

A two-dimensional image, such as a photograph or a cephalogram of a three-dimensional object, is unlikely to provide all the information needed [1]. Therefore, 3D evaluation techniques are available that have been shown to be very accurate in representing 3D structures. Three-dimensional stereophotogrammetry is the preferred technique for evaluating the symmetry of facial soft tissue [1,7].

Class II patients undergoing orthognathic surgery have never been systematically evaluated for facial soft tissue outcomes beyond 6 months postoperatively using three-dimensional stereophotogrammetry. Better understanding of facial soft tissue results following surgical procedures may have an important clinical impact and improve patient counseling [10]. Other studies have often emphasized the lack of sufficient symmetry in facial soft tissue [11].

Only a few reports have described the exact changes in facial soft tissue and the long-term follow-up (>6 months) after orthognathic surgery in Class II patients [12]. Due to differences in methodology and materials employed, the results of these studies are inconsistent [7]. Therefore, the aim of the present study was to evaluate the changes in facial soft tissue asymmetry 12 months after orthognathic surgery in Class II patients with skeletal deformities using 3D stereophotogrammetry.

## 2. Materials and Methods

### 2.1. Study Design and Samples

This prospective, longitudinal cohort study enrolled 54 Caucasian patients (32 women and 22 men) with Class II skeletal malocclusion. Each patient had undergone pre- and post-orthodontic treatment and orthognathic surgery between 2012 and 2022 at the Department of Orthodontics, Institute of Stomatology, Rīga Stradiņš University (Riga, Latvia). The mean age was 33.2 years (age range, 22–60 years; standard deviation (SD), 7.72). The follow-up period was 12 months.

Two experienced oral and maxillofacial surgeons performed all orthognathic surgeries. The clinical findings determined the type of surgery: single-jaw surgery, bilateral sagittal split osteotomy (BSSO)-24 (44.4%), bimaxillary surgery, Le Fort I osteotomy in combination with BSSO-30 (55.6%).

Three-dimensional images were acquired for all patients before surgery (T0), 6 months after (T1), and 12 months (T2) after surgery using stereophotogrammetry. Differences between groups were examined at three time points. Groups were compared for time of image acquisition, facial area (whole face, upper, middle, and lower facial thirds), type of surgery, sex, and pogonion point (pogo) deviation.

### 2.2. Ethical Considerations and Study Registration

This research adhered to internationally accepted ethical guidelines and was carried out with the permission of the Ethics Committee of the Rīga Stradiņš University (RSU Approval Number: E-9(2); date, 26 April 2012). This study was carried out by following the ethical criteria of the Declaration of Helsinki and Current Controlled Trials ISRCTN14166937. Each patient provided informed consent, and participation was voluntary. Data confidentiality was ensured.

### 2.3. Inclusion and Exclusion Criteria

The patient selection criteria were as follows: Class II skeletal malocclusion; no history of trauma or craniofacial syndromes, no previous orthodontic treatment or orthognathic surgery; pre- and postoperative stereophotogrammetry records at three time points.

The following exclusion criteria were applied: patients with syndromes or craniofacial anomalies or with cleft lip and/or palate; patients who did not have a 3D image or patients with beards and/or mustaches; patients with a deformed 3D image.

### 2.4. Stereophotogrammetry System and Image Acquisition

Three-dimensional photographs were obtained from each patient using the 3dMD Trio stereophotogrammetry system (3dMD LLC, Atlanta, GA, USA). The facial surface images were recorded at T0, T1, and T2 time points.

During this 3D imaging procedure, an adjustable chair was used for the patients. Participants were instructed to maintain a neutral facial expression, with their teeth in light occlusal contact and their head positioned naturally, as described in previous studies [13]. The head and neck areas were recorded with the 3dMD system. The exposure was repeated if the patient moved. Two trained photographers took all 3D photographs.

These scanned images were then exported to a workstation using 3dMD Vultus version 2.5.0.1 (3dMD LLC) and edited using 3dMD Patient version 4.1 software (3dMD LLC). Manual adjustments were performed to delineate the 3D surface boundaries, removing irrelevant or interfering areas. For instance, the shoulders, neck, and ears were manually removed from the 3D scans. These areas are vulnerable to error and may affect the quantification of asymmetry [13].

### 2.5. Quantification of Asymmetry

3dMD Vultus version 2.5.0.1 (3dMD, LLC) was used for displaying the 3D models in four separate view windows [14].

**Figure 1:**
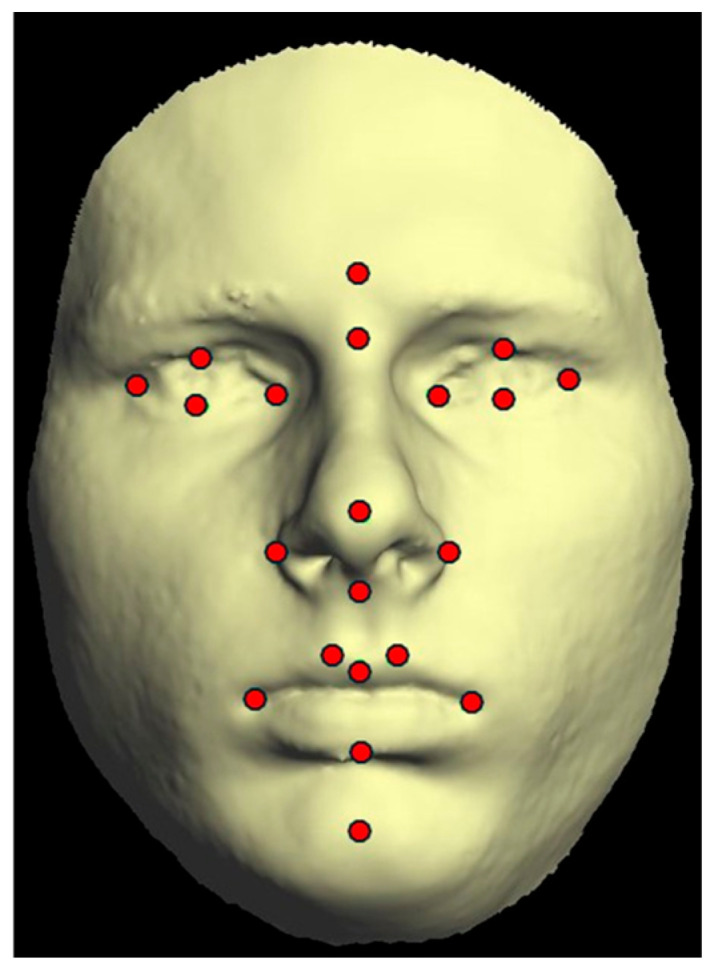
Anthropometric landmarks identified on 3D frontal monochrome images (*n* = 21): Glab, glabella; Nasi, soft tissue nasion; En_R, right endocanthion; En_L, left endocanthion; Ex_R, right exocanthion; Ex_L, left exocanthion; psLe, left palpebrale superius; psRi, right palpebrale superius; piRi, right palpebrale inferius; piLi, left palpebrale inferius; Pro, pronasale; Subn, subnasale; AL_R, right nasal ala; AL_L, left nasal ala; LaSu, labrale superius; LaIn, labrale inferius; chRi, right cheilion; chLe, left cheilion; cphR, crista philtri right; cphL, crista philtri left; Pogo, soft tissue pogonion.

A single operator digitally identified and marked 21 anthropometric landmarks for every 3D facial image at three specific time points (Figure 1). From the software, each landmark location was determined in three spatial axes: x, y, and z. These landmarks were chosen in accordance with methodologies outlined in prior studies [15,16,17]. Appendix A (Table A1) provides a detailed overview of the landmarks and their respective definitions.

**Figure 2:**
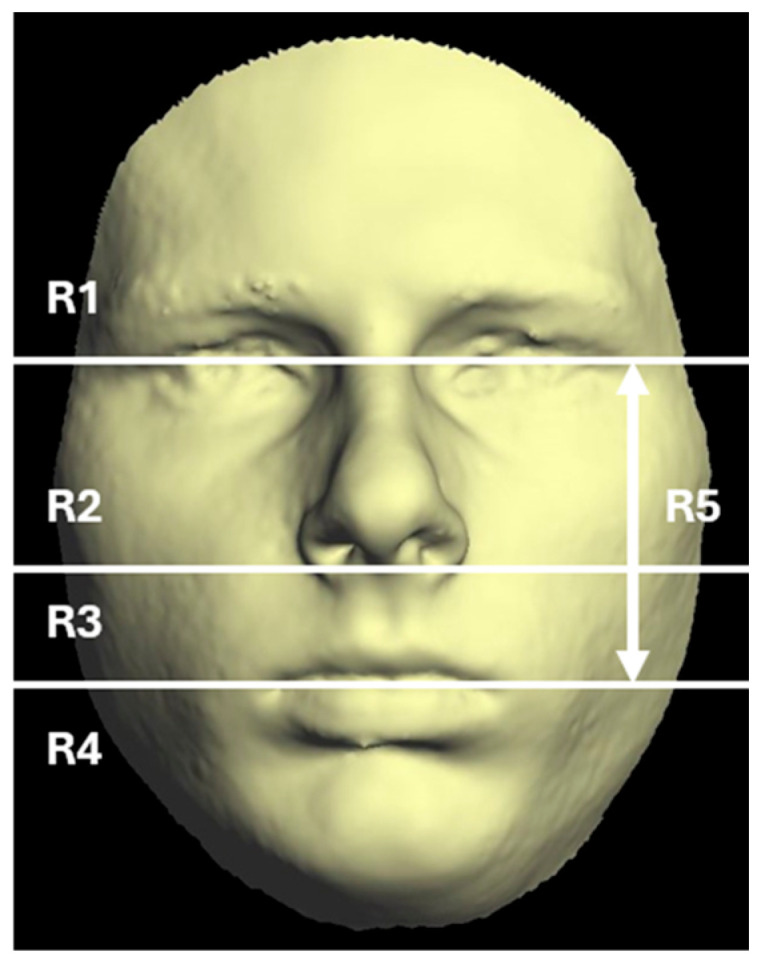
The facial surface was separated into five anatomical regions: region 1 (R1), the part of the face above the mid-eye line; region 2 (R2), between subnasale (sn) and the mid-eye line; region 3 (R3), between the mid-lip line (chLe–chRi) and subnasale; region 4 (R4), the part of the face below the mid-lip line (chin zone); region 5 (R5), between the mid-lip line (chLe–chRi) and mid-eye line.

After the calibration process, all landmarks (7 bilateral and 7 medial points) were assigned across the entire patient cohort. A mirroring method applied to 3D facial models was employed to quantify asymmetry in each patient [15]. Rapidform 2006 software (Geomagic, Rock Hill, SC, USA) was used to perform all analytical procedures. As previously described [18], the facial position was standardized prior to analysis. A mirrored replica of each facial surface was then created and aligned to the original using a best-fit superimposition technique. Quantitative analysis included calculating the mean and maximum deviations between the original and mirrored facial surfaces [13,15,19,20,21,22]. Each original facial surface was divided into five regions (Figure 2).

Chin asymmetry was measured from the 3D facial model using the Chin Volume Asymmetry Score (CVAS) [23]. The area of the chin was segmented into two solid shapes and the volume of each was calculated [24]. As previously described, the degree of asymmetry was determined based on the volumetric ratio of the respective facial segments [24,25]. The CVAS was calculated as the ratio between larger to smaller regional volumes [23].

The horizontal symmetry of the face was analyzed using linear parameters. Based on the lateral deviation of the pogonion from the midsagittal plane, patients were classified into three asymmetry groups: mild (0–2 mm), moderate (3–5 mm), and severe (>5 mm).

### 2.6. Error of Method Analysis

The initial phase of the study involved assessing the repeatability of the operator in identifying anthropometric landmarks on 3D facial surfaces. A single operator performed the landmark identification. A training sample of 15 facial surfaces was randomly selected across all groups and digitally marked with all landmarks. After an interval of 2 weeks, the measurement session was repeated under the same conditions. Landmark coordinates were extracted from the software and documented in a Microsoft Excel spreadsheet for further analysis. Statistical verification procedures were applied [26] and classified into categories (<0.5, 0.5 ≤ 1.0, and >1.0 mm).

### 2.7. Statistical Analysis

Statistical computations were conducted using R version 3.6.1 software (R Foundation for Statistical Computing, Vienna, Austria). The intra-operator reliability for the evaluator has been quantified in a previous study [24].

All groups were assessed for normality using Q-Q plots and supplemented with Lilliefors and Kolmogorov–Smirnov tests in cases when normality could not be assumed unequivocally. A Greenhouse–Geisser-adjusted repeated measures ANOVA was utilized to compare intra-group variations across conditions. In case of statistically significant differences indicated by the repeated measures ANOVA, a paired *t*-test was used for the corresponding groups for pairwise comparisons. Statistical significance was set at a *p*-value of less than 0.05.

## 3. Results

**Figure 3:**
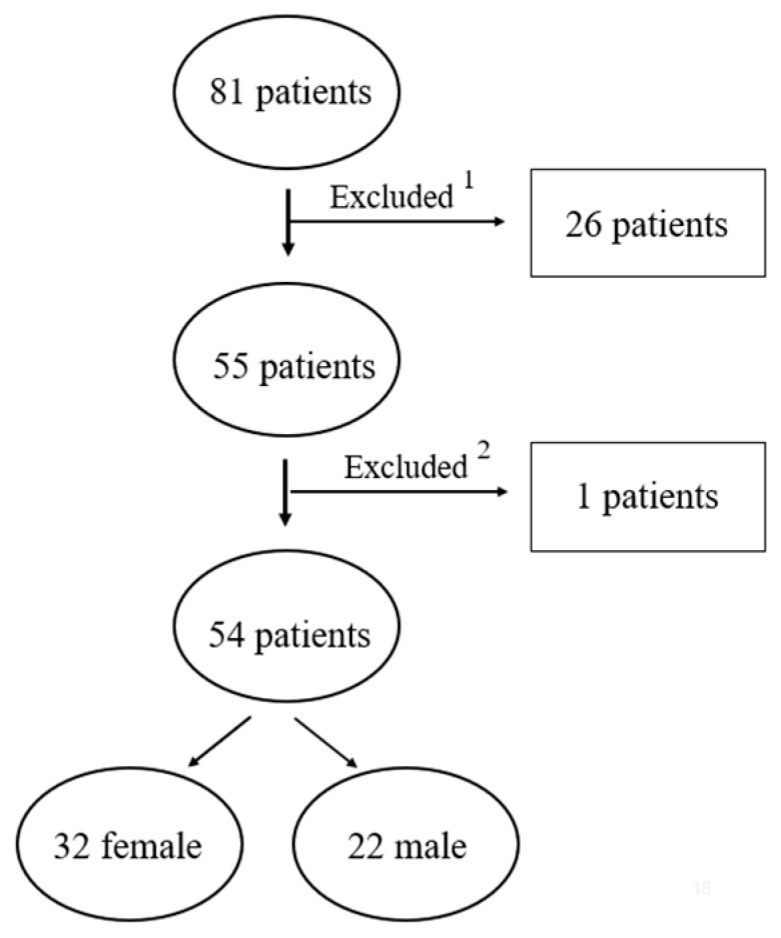
Patient enrollment flowchart detailing inclusion and exclusion processes. ^1^ Absence of 3D facial images at any time point or presence of image artifacts/defects. ^2^ Image distortion resulting from facial hair rendered one of the three patient scans unusable, as it could lead to inaccurate measurements.

The study cohort comprised 81 patients diagnosed with a skeletal Class II malocclusion. The study population consisted of patients who had undergone orthodontic and orthognathic surgery at the Institute of Stomatology, Rīga Stradiņš University, Latvia. Twenty-seven patients (33% of the total sample) were excluded from subsequent analysis (Figure 3). A total of 54 patients fulfilled the established criteria and were selected for subsequent 3D image evaluation (Table 1).

### 3.1. Landmark Accuracy

The mean error in anthropometric landmark identification was 0.3 mm, with a range of 0.08 to 0.58 mm. Intra-operator consistency for facial landmark identification demonstrated high reliability, with values ranging between 0.859 and 0.998, as previously reported [24]. The 95% confidence interval indicated no significant difference in landmark position accuracy between operator measurements taken at 2-week intervals.

### 3.2. Primary Outcome

**Figure 4:**
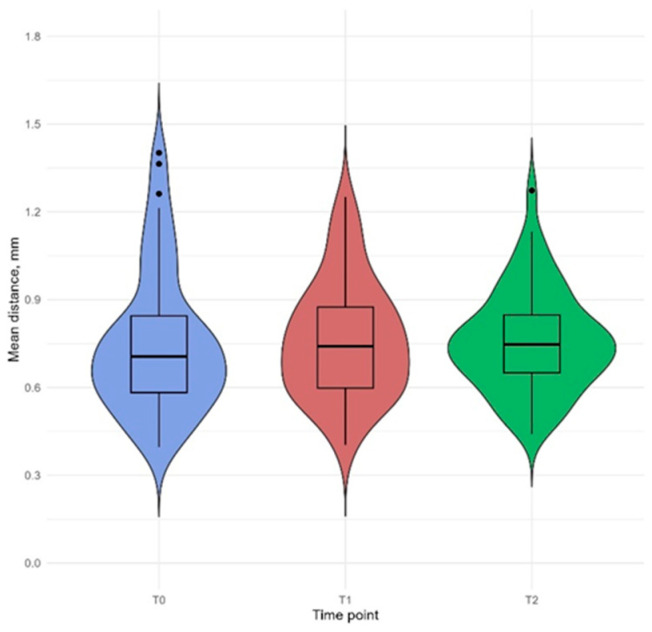
Box plot illustrates the mean distance (MnD) for the whole face at three time points: pre-surgery (T0), 6 months post-surgery (T1), and 12 months post-surgery (T2). Data points falling outside the interquartile range are depicted as individual markers and considered potential outliers.

The assessment of whole-face asymmetry indicated a minor difference in mean distance values, with median MnD increasing from 0.70 mm at T0 to 0.74 mm at T1 (Figure 4).

However, a statistically significant difference was not observed at any of the time points. There was also no statistically significant difference in maximum distance (MD) between T0 (median 3.29 mm) and T2 (median 3.43 mm) time points (Table 2).

**Figure 5:**
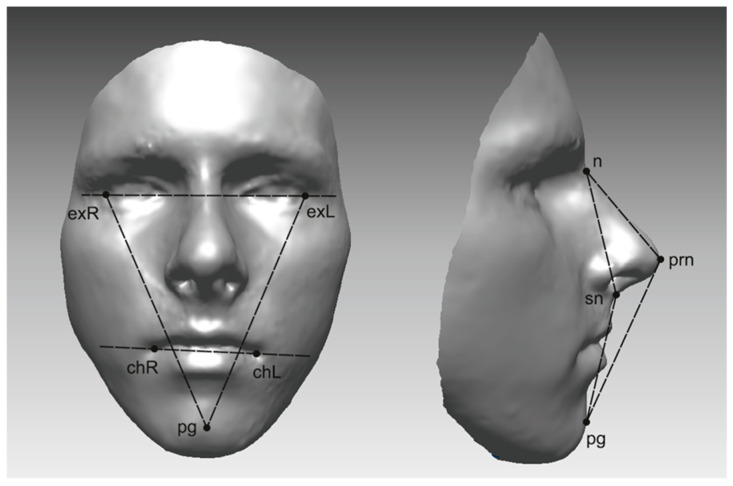
The 3D angle measurements of the whole face (monochrome images): 1. n-sn-pg; 2. n-prn-pg; 3. exR-exL-pg; 4. exL-exR-pg; 5. exRexL-chRchL. Frontal image: exR, right exocanthion; exL, left exocanthion; chR, right cheilion; chL, left cheilion; pg, soft tissue pogonion. Profile image: n, soft tissue nasion; prn, pronasale; sn, subnasale.

The 3D angle measurements of the whole face received more attention (Table 3) (Figure 5). The 3D measurements n-sn-pg and n-prn-pg were compared between T0 versus T1 and T2 time points and showed statistically significant differences (*p* < 0.05). The mean 3D angles between exR-exL-pg and exL-exR-pg at T0 were 65.98° and 66.24° degrees, respectively, but there was no statistically significant change in these values after surgery. The 3D angle between exRexL and chRchL represented a slight asymmetry of the lip line of 2.36°. All linear parameters did not show differences of more than 1 mm.

### 3.3. Subgroups

No statistically significant facial asymmetry was found in any region of the face at any time point (Figure 2). Before surgical intervention, the lower and middle facial regions exhibited the highest asymmetry, with mean distances of 3.26 mm (region 4) and 2.56 mm (region 5), respectively.

A comparison between the sexes revealed no statistically significant differences in facial asymmetry at any time point and in any region of the face. Analysis of asymmetry outcomes demonstrated consistent correlation trends in both groups (Table 4). The soft tissue asymmetry of male faces was observed to be higher than female faces prior to surgery. However, this difference was not found to be statistically significant (MnD T0: 0.717 mm in male patients versus 0.671 mm in female patients).

No statistically significant differences were found between 3D angular parameters and sex (Table 5). However, there were statistical differences in the 3D angles of n-sn-pg and n-prn-pg between time points in both groups (*p* < 0.05).

The patients were classified based on the type of surgical procedure. No statistically significant differences were observed in either the MnD or MD between the groups at any time point. There was a greater propensity for facial asymmetry in the bimaxillary surgical group, though this difference lacked statistical significance. The MD between the facial shells before surgery was recorded as 3.56 mm in the bimaxillary group and 3.23 mm in the single-jaw group (Table 6). The 3D angle measurements of n-sn-pg and n-prn-pg were statistically different between time points in both groups (*p* < 0.05).

### 3.4. Deviation of the Pogonion Point

**Figure 6:**
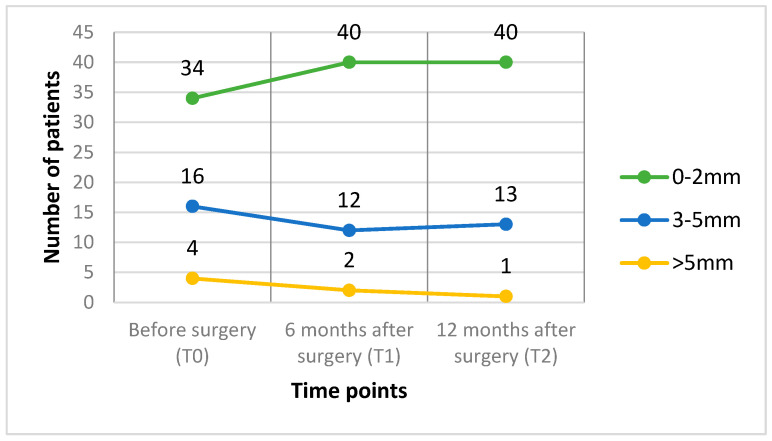
Changes in the distribution of patients across asymmetry groups at three time points: pre-surgery, 6 months post-surgery, and 12 months post-surgery. Patients were classified based on the deviation of the pogonion point from the midsagittal plane into three categories: mild (0–2 mm), moderate (3–5 mm), and severe (>5 mm) asymmetry.

The asymmetry group distribution showed fluctuations at different time points (Figure 6). The number of patients exhibiting mild asymmetry following surgical intervention increased.

No statistically significant differences were identified in the MnD or MD between any time point. There were statistical differences in 3D angle measurements of n-sn-pg and n-prn-pg between time points in the mild asymmetry group (*p* < 0.05) (Table 7).

### 3.5. Chin Volume Asymmetry Score

**Figure 7:**
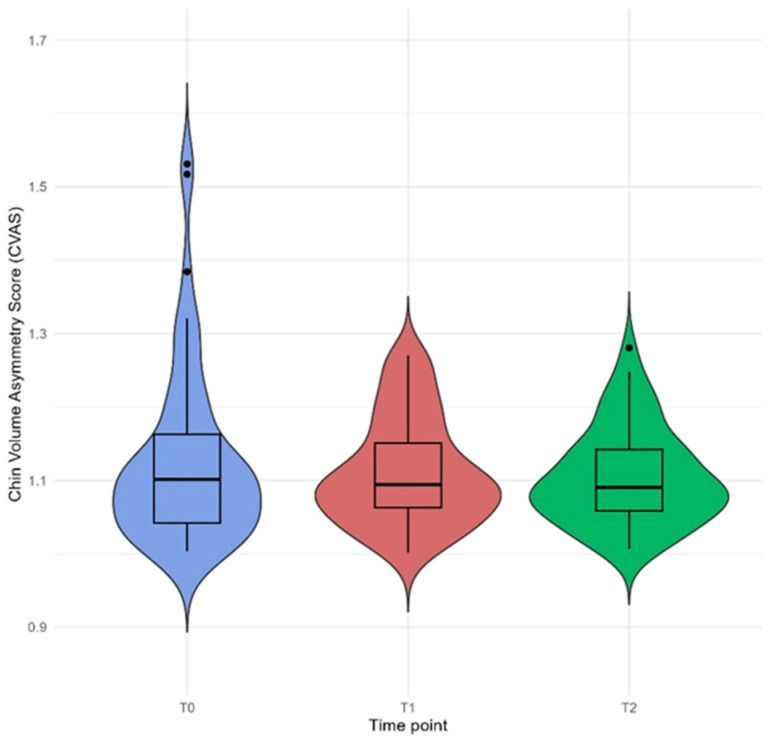
Box plot illustrates the Chin Volume Asymmetry Score (CVAS) at three time points: pre-surgery (T0), 6 months post-surgery (T1), and 12 months post-surgery (T2). Data points falling outside the interquartile range are displayed as individual markers and considered potential outliers. Three potential outliers were identified at T0 and one at T2. The CVAS has a minimum value of 1, with higher values indicating greater facial asymmetry.

Changes in CVAS across the three assessed time points, surgery groups, or sex groups were not statistically significant (Figure 7) (Table 8). The preoperative score was higher in the female group and patients undergoing bimaxillary surgery than in the male group and patients undergoing single-jaw surgery, but the difference was not statistically significant. Postoperative CVAS values at the T2 time point were similar in all groups.

## 4. Discussion

The present report has focused on the issue of facial soft tissue asymmetry, which has a more direct impact on esthetic considerations than facial hard tissue asymmetry. Patients desire an enhanced esthetic appearance, and they seek orthodontic and orthognathic treatment [3,27,28].

In orthodontics and oral surgery, 3D photogrammetry is now a routine part of patient assessment [21]. The application of the 3D stereophotogrammetry method enables the surgeon to conduct a more precise patient analysis [28]. The method represents an advanced non-invasive and non-contact surface scanning technique capable of reconstructing 3D shape structures from plain photographs, thus providing a valuable radiation-free tool for researchers [1,3,27,29,30]. In this study, a surface-based method was employed for the analysis of facial asymmetry.

The correlation between facial asymmetry and skeletal class is controversial. Some authors claim that patients with skeletal Class I, II, and III are equally likely to have facial asymmetry [3,31] while other authors have reported that asymmetry is most associated with Class III and less frequently with Class II [4]. A previous study [24] involving patients with skeletal Class III deformities showed that almost 40% of the patients had an asymmetry of more than 2 mm, but in the present study, this value was 37% for patients with skeletal Class II.

There is no consensus on the part of the face with the most asymmetry in patients with Class II deformities. The findings of the present study align with those of previous research, which also demonstrated greater asymmetry in the lower facial region [5,10,13,29,31]. Facial asymmetry appears to be most common in patients with a vertical growth pattern [4].

The most notable feature of facial asymmetry is the deviation of the chin [10]. In previous studies, a deviation of the chin was present in 74% of the patients with facial asymmetry [4]. In agreement with this, the present study showed that nearly 63% of patients had mild asymmetry (0–2 mm) before surgery: 30% had moderate asymmetry (3–5 mm) and only 7% had severe asymmetry (>5 mm).

Facial asymmetry is similar in males and females, according to many studies [3], but this remains controversial [3,29]. In the present study, sex was not significantly associated with facial asymmetry. Males showed larger mean face asymmetry values and change in face symmetry than females [3,32], but these differences did not reach statistical significance, which is consistent with previous studies [15,29,32]. There was no significant difference in facial symmetry between the thirds of the face when controlling for sex.

Some studies have recommended orthognathic surgery to achieve optimal facial symmetry when the mandibular deviation is greater than 2 mm and recommended bimaxillary surgery instead of mandibular surgery alone [3,10]. Our current data revealed that bimaxillary surgery was the most common type (55.6%). However, chin advancement was not performed in any of the cases. In the literature, genioplasty is often reported to be the final step for refinement of the chin position [10]. Previous studies have shown that most Class II patients have undergone bimaxillary surgery followed by genioplasty to further improve their facial profile and symmetry [2]. Chin asymmetry improved significantly after the operation, but some residual asymmetry remained, indicating the difficulty in recognizing the facial midline during the operation or its recurrence after the operation [1,10].

Facial symmetry is predominantly influenced by skeletal structure. However, facial soft tissue plays a pivotal role in shaping the facial contours, which ultimately determines overall facial symmetry [3]. In addition, prior research has indicated that soft tissue exhibits a greater degree of symmetry compared to hard tissue. These differences may be attributed to variations in the thickness of the soft tissue [3,12]. The degree of soft tissue response following orthognathic surgery and the extent of symmetry changes remain unpredictable, particularly in the maxillary and midfacial regions [27]. The correlation between hard and soft tissue changes in the lower face was found to be relatively high, thereby facilitating more predictable results [2,27].

Due to the bone changes induced by the surgery, the postoperative improvements of the face are more pronounced in the first 6 months than the changes from T1 to T2. However, it should be noted that correcting facial soft tissue asymmetries is very challenging [12,30,33]. Therefore, patients should be informed that some asymmetry may remain after orthognathic surgery despite successful correction of the bone deformity [4,12].

The findings emphasize the temporal dynamics of postoperative healing, particularly the progressive changes in soft tissue swelling, remodeling, and repositioning [12,34]. Gill et al. (2017) [12] reported that a residual facial edema can persist at up to 10–15% of its initial volume for between 6 and 12 months postoperatively. Accordingly, objective evaluation of soft tissue outcomes is not recommended prior to six months after surgery, as earlier assessments may not reflect the final morphological state [12,35]. Evaluations conducted at 12 months are generally considered more reliable, representing a stabilized phase of soft tissue adaptation [36].

The present study has several limitations. First, there was no comparative analysis between primary soft tissue changes and actual skeletal movement observed in a cone-beam computed tomogram (CBCT). The impact of the underlying skeletal structure on facial asymmetry was not investigated. Second, the considerable age range precluded the possibility of establishing a correlation between asymmetry and age. The study is limited by its relatively small sample size and the fact that it was conducted at a single center. There was a discrepancy in the number of participants in the sexes, with a relatively low proportion of men. Further, the groups defined by surgical type were not homogeneous. This study did not include an examination of other biological factors, such as lip thickness, tonality, and volume, that may affect facial symmetry.

This study is unique in its assessment of facial soft tissue asymmetry in a sizable cohort of Class II patients with skeletal deformity who underwent 3D imaging following orthognathic surgery at three distinct time points using a 3D stereophotogrammetry technique. A longitudinal study employing the 3D stereophotogrammetry technique is required to further ascertain long-term alterations in the asymmetry of facial soft tissue after surgery and to evaluate the stability and progression of soft tissue adaptations.

## 5. Conclusions

A reduction in the asymmetry of facial soft tissue was observed in patients with Class II malocclusion following orthognathic surgery. The results of the study indicate that asymmetry of facial soft tissue may undergo a change within a 6-month period following surgery. It is recommended that the final response of facial soft tissues be assessed no earlier than six months post-surgery, as this period allows for sufficient stability to be attained. Further research is required to examine the long-term alterations in facial asymmetry (beyond 12 months) following orthognathic surgery in patients with Class II malocclusion. Future studies should employ the 3D stereophotogrammetry technique, with a comparison made between this and the skeletal movements observed on CBCT.

## Figures and Tables

**Table 1:** Selected data and distribution of patients (*n* = 54).

Age (Years)	
Range	22–60
Mean ± SD	33.2 ± 7.72
Sex, *n* (%)	
Women	32 (59.3%)
Men	22 (40.7%)
Surgery type	
Bimaxillary	30 (55.6%)
BSSO	24 (44.4%)

**Table 2:** Descriptive statistics and comparative analysis of mean and maximum distances representing whole-face and regional facial asymmetry at three time points: pre-surgery (T0), 6 months post-surgery (T1), and 12 months post-surgery (T2). *p*-values < 0.05 (*) were considered statistically significant.

	T0	T1	T2	
	Median (mm)	Median (mm)	Median (mm)	*p*-Value
Whole face				
MnD	0.71	0.74	0.75	0.769
MD	3.29	3.26	3.43	0.659
Region 2				
MnD	0.55	0.61	0.58	0.151
MD	2.19	2.39	2.35	0.337
Region 3				
MnD	0.70	0.77	0.77	0.696
MD	2.49	2.69	2.83	0.636
Region 4				
MnD	0.96	1.07	1.04	0.650
MD	3.27	3.11	3.36	0.375
Region 5				
MnD	0.60	0.65	0.68	0.497
MD	2.56	2.86	2.91	0.433

Region 2 (R2), part of the face between the subnasale (sn) and mid-eye line; Region 3 (R3), part of the face between the mid-lip line (chL-chR) and subnasale; Region 4 (R4), part of the face below the mid-lip line (chin zone); Region 5 (R5), part of the face between the mid-lip line (chL-chR) and mid-eye line. MnD, mean distance; MD, Maximum distance.

**Table 3:** Descriptive statistics and comparisons of 3D angle measurements of the whole face at all three time points: pre-surgery (T0), 6 months post-surgery (T1), and 12 months post-surgery (T2). *p*-values < 0.05 (*) were considered statistically significant.

3D Angle Measurements	T0	T1	T2	
	Median (Degrees)	Median (Degrees)	Median (Degrees)	*p*-Value
n-sn-pg	154.24	157.13	158.80	0.001 *
n-prn-pg	122.33	125.20	125.39	0.001 *
exR-exL-pg	65.98	65.84	65.89	0.351
exL-exR-pg	66.23	66.31	66.39	0.790
exRexL-chRchL	2.37	2.30	2.40	0.729

**Table 4:** Descriptive statistics and comparisons of facial asymmetry between different sex groups in terms of mean and maximum distances and at three pre-surgery time points: before surgery (T0), 6 months post-surgery (T1), and 12 months post-surgery (T2). *p*-values < 0.05 (*) were considered statistically significant.

Sex	T0	T1	T2		
	Median (mm)	Median (mm)	Median (mm)	*p*-Value	*p*-Value (Between Sex)
Male					
MnD	0.72	0.77	0.77	0.929	
MD	3.68	3.39	3.46	0.429	0.945
Female					
MnD	0.67	0.70	0.73	0.772	
MD	2.99	2.97	3.41	0.328	

MnD, mean distance; MD, maximum distance.

**Table 5:** Descriptive statistics and comparisons of facial asymmetry between different 3D angular parameters and sex at three time points: pre-surgery (T0), 6 months post-surgery (T1), and 12 months post-surgery (T2). *p*-values < 0.05 (*) were considered statistically significant.

Sex	T0	T1	T2	
	Median (Degrees)	Median (Degrees)	Median (Degrees)	*p*-Value
Male				
n-sn-pg	153.32	156.12	157.75	0.001 *
n-prn-pg	120.73	124.00	123.75	0.001 *
Female				
n-sn-pg	154.98	159.22	160.49	0.001 *
n-prn-pg	122.68	126.41	125.95	0.001 *

**Table 6:** Descriptive statistics and comparison of facial asymmetry between surgery type groups in terms of mean and maximum distances and 3D angular parameters and at different time points: pre-surgery (T0), 6 months post-surgery (T1), and 12 months post-surgery (T2). *p*-values < 0.05 (*) were considered statistically significant.

	T0	T1	T2		
Surgery Type	Median (mm)	Median (mm)	Median (mm)	*p*-Value	*p*-Value (Between Operation Types)
BSSO					
MnD	0.67	0.72	0.71	0.960	
MD	3.24	3.15	3.08	0.450	
n-sn-pg	154.09	157.05	158.19	0.001 *	
n-prn-pg	121.90	124.04	124.17	0.001 *	0.756
LF + BSSO					
MnD	0.72	0.78	0.77	0.692	
MD	3.56	3.39	3.64	0.369	
n-sn-pg	154.41	157.29	158.93	0.001 *	
n-prn-pg	122.78	126.41	126.61	0.001 *	

LF, Le Fort I osteotomy; LF + BSSO, Le Fort I osteotomy and bilateral sagittal split osteotomy; MnD, mean distance; MD, maximum distance.

**Table 7:** Descriptive statistics and comparisons of asymmetry groups (deviation of the pogonion point) in terms of mean and maximum distances in region 4; 3D angle measurements in every time point (before (T0), 6 months after surgery (T1), and 12 months after surgery (T2)). A *p*-value of <0.05 (*) was considered statistically significant.

Region 4	T0	T1	T2	
	Median (mm)	Median (mm)	Median (mm)	*p*-Value
0–2				
MnD	0.65	0.70	0.73	0.936
MD	2.79	2.89	3.20	0.160
3–5				
MnD	0.77	0.89	0.78	0.868
MD	3.79	4.08	3.88	0.777
>5				
MnD	1.18	1.07	0.94	-
MD	4.94	4.96	4.88	-
0–2				
n-sn-pg	154.24	157.51	158.73	0.001 *
n-prn-pg	121.94	124.89	124.73	0.001 *
3–5				
n-sn-pg	154.72	156.55	158.67	0.199
n-prn-pg	123.36	124.96	126.01	0.114
>5				
n-sn-pg	153.37	164.07	161.12	-
n-prn-pg	123.65	130.99	127.84	-

MnD, mean distance; MD, maximum distance.

**Table 8:** Chin Volume Asymmetry Score (CVAS) comparison across (a) the three time points—pre-surgery (T0), 6 months post-surgery (T1), and 12 months post-surgery (T2); (b) sex groups; and (c) surgical procedure groups. The CVAS has a minimum value of 1, with higher values indicating greater asymmetry.

	T0	T1	T2	
CVAS	Median (mm)	Median (mm)	Median (mm)	*p*-Value
(a) Time	1.10	1.09	1.09	0.901
(b) Sex				
Male	1.09	1.08	1.09	0.555
Female	1.11	1.10	1.09	0.186
(c) Surgery type				
LF + BSSO	1.11	1.10	1.09	0.350
BSSO	1.08	1.05	1.09	0.273

LF, Le Fort I osteotomy; LF + BSSO, Le Fort I osteotomy and bilateral sagittal split osteotomy.

## Data Availability

All data are available in the main text. The protocols and the anonymized datasets generated and/or analyzed during the current study are available from the corresponding author on reasonable request. The patient’s photos and any other identifying information cannot be shared.

## Abbreviations

The following abbreviations are used in this manuscript:

3D	Three-dimensional
BSSO	Bilateral sagittal split osteotomy
CVAS	Chin Volume Asymmetry Score
CBCT	Cone-beam computed tomogram
MnD	Mean distance
MD	Maximum distance
SD	Standard deviation
T0	Before surgery
T1	6 months after surgery
T2	12 months after surgery

## Appendix A

**Table A1:** Definitions of facial landmarks in 3D (three-dimensional) images.

Landmark	Abbreviation	Definition
Glabella	Glab	The most anterior midpoint on the fronto-orbital soft tissue contour
Nasion	Nasi	The deepest point of nasal bridge (of the frontonasal suture)
Endocanthion	EN_R	The soft tissue point located at the inner commissure of each eye fissure
Exocanthion	EX_R	The soft tissue point located at the outer commissure of each eye fissure
Endocanthion	EN_L	The soft tissue point located at the inner commissure of each eye fissure
Exocanthion	EX_L	The soft tissue point located at the outer commissure of each eye fissure
Palpebrale superius	psLe	The highest point in the mid-position of the free margin of each upper eyelid
Palpebrale superius	psRi	The highest point in the mid-position of the free margin of each upper eyelid
Palpebrale inferius	piRi	The lowest point in the mid-position of the free margin of each lower eyelid
Palpebrale inferius	piLe	The lowest point in the mid-position of the free margin of each lower eyelid
Pronasale	Pron	The most anterior midpoint of the nasal tip
Subnasale	Subn	The midpoint on the nasolabial soft tissue contour between the columella crest and the upper lip
Alare right	AL_R	The most lateral point on right alar contour
Alare left	AL_L	The most lateral point on left alar contour
Labiale superius	LaSu	The midpoint of the vermilion line of the upper lip
Labiale inferius	LaIn	The midpoint of the vermilion line of the lower lip
Christa philtri left	cphL	The point at left crossing of the vermilion line and the elevated margin of the philtrum
Christa philtri right	cphR	The point at right crossing of the vermilion line and the elevated margin of the philtrum
Cheilion left	chLe	The point located at the left labial commissure
Cheilion right	chRi	The point located at the right labial commissure
Pogonion	Pogo	Most anterior midpoint of the chin
